# Prospective Clinical Evidence on Artificial Intelligence-Enabled Augmented and Mixed Reality in Robot-Assisted Prostate and Kidney Cancer Surgery: A Systematic Review

**DOI:** 10.7759/cureus.116810

**Published:** 2026-09-23

**Authors:** Gregory Harrison

**Affiliations:** 1 Urology, Royal London Hospital, London, GBR

**Keywords:** artificial intelligence in surgery, augmented reality surgical navigation, mixed reality, robot-assisted partial nephrectomy, robot-assisted radical prostatectomy, systematic literature review

## Abstract

Intraoperative digital guidance is increasingly being integrated into robot-assisted radical prostatectomy and robot-assisted partial nephrectomy to improve oncological precision while preserving functional outcomes. The supporting clinical evidence remains limited and heterogeneous. This systematic review, conducted in accordance with the Preferred Reporting Items for Systematic Reviews and Meta-Analyses (PRISMA) 2020 guidelines and prospectively registered in PROSPERO (CRD420261400733), searched PubMed, Ovid Embase, and the Cochrane Library for English-language studies published between 1 January 2025 and 20 May 2026. Eligible studies were prospective investigations involving adults undergoing robot-assisted radical prostatectomy or robot-assisted partial nephrectomy for primary urological malignancy, evaluating intraoperative augmented reality, mixed reality, artificial intelligence (AI)-assisted pathology, and related image-guided technologies with at least one patient-level clinical or diagnostic endpoint. Narrative synthesis was performed because of substantial heterogeneity, and risk of bias was assessed using design-specific tools. Of the 131 records screened, 93 were excluded at the title and abstract screening stage, 22 reports underwent full-text eligibility assessment after removal of duplicates, 16 reports were excluded at the full-text eligibility assessment stage, and six studies were included in the narrative synthesis. In radical prostatectomy, 3D AI-augmented reality guidance and AI-enhanced stimulated Raman histopathology showed promising signals for surgical margin control, adjuvant treatment, continence, and intraoperative margin assessment. In partial nephrectomy, mixed reality improved warm ischaemia time and clamping strategy, while 3D image-guided surgery demonstrated acceptable medium-term oncological and functional outcomes. Overall, prospective evidence suggests that intraoperative digital guidance may improve selected oncological, functional, technical, and diagnostic outcomes in robotic urological surgery, but larger multicentre comparative trials with standardised endpoints and longer follow-up are required.

## Introduction and background

Robot-assisted surgery is now embedded in contemporary urological oncology, particularly in radical prostatectomy and partial nephrectomy. In radical prostatectomy, the tension between nerve preservation and margin control remains one of the defining intraoperative challenges, as positive surgical margins may increase the risk of biochemical recurrence and the need for adjuvant treatment, whereas excessive dissection may compromise continence and erectile recovery [1-5]. In partial nephrectomy, the parallel challenge is to achieve complete tumour excision while minimising warm ischaemia, avoiding preventable complications, and preserving postoperative renal function [6,7].

These pressures have accelerated interest in digital intraoperative support technologies. Augmented reality (AR) and mixed reality (MR) aim to improve spatial orientation by overlaying reconstructed anatomy or tumour maps onto the operative workflow, while AI-assisted pathology platforms seek to deliver near-real-time tissue interpretation for intraoperative decision support. Three-dimensional image guidance has also become increasingly sophisticated, but the underlying technologies differ materially in mechanism, maturity, and evidentiary expectations. Not every tool described as *AI-enabled* actually functions as an autonomous or inferential AI platform; in several instances, the clinical value appears to derive primarily from image registration, structured visualisation, or real-time display rather than from machine-learning decision-making [8-10].

This distinction matters because the current literature spans randomised interventional studies, non-comparative feasibility studies, prospective registries, and diagnostic validation reports. Recent high-level work in intraoperative prostate cancer surgery, including randomised evidence for NeuroSAFE-guided nerve-sparing, has shown that intraoperative decision support can translate into improved functional outcomes when deployed within robust clinical pathways [3]. By contrast, many newer AR, MR, and AI-labelled technologies remain at a much earlier stage of evaluation [8-10].

The primary objective of the study was to identify and narratively synthesise prospective clinical studies of intraoperative AI-enabled AR, optical histology, MR, and related digital guidance technologies used during robot-assisted radical prostatectomy and robot-assisted partial nephrectomy. A secondary objective was to examine whether self-applied *AI* labelling corresponded to the underlying technical architecture of the included systems.

## Review

Protocol and reporting standard

This review was conducted in accordance with the PRISMA 2020 statement for systematic reviews [11]. The protocol was prospectively registered in the International Prospective Register of Systematic Reviews (PROSPERO; CRD420261400733). The registered review title was Prospective Evidence on Intraoperative AI-Enabled Augmented Reality, Optical Histology, and Mixed-Reality Navigation in Robot-Assisted Urological Oncology Surgery.

Information sources and search strategy

PubMed, Ovid Embase, and the Cochrane Library were searched for studies published between 1 January 2025 and 20 May 2026. The search strategy combined controlled vocabulary and free-text terms across three conceptual domains: surgical procedure, intraoperative technology, and clinical context.

The first domain covered the surgical procedure: robot-assisted radical prostatectomy, RARP, robot-assisted partial nephrectomy, RAPN, robotic prostatectomy, and robotic nephrectomy. The second covered the intraoperative technology: AR, MR, AI, machine learning, deep learning, stimulated Raman histology, optical histology, 3D model, three-dimensional navigation, and intraoperative guidance. The third covered the clinical context: prostate cancer, renal cell carcinoma, surgical margin, positive surgical margin, warm ischaemia, and trifecta. These three domains were combined with the Boolean operator AND, and terms within each domain were combined with OR.

Study selection

Studies were eligible if they enrolled adults (≥18 years) undergoing robot-assisted radical prostatectomy or robot-assisted partial nephrectomy for histologically confirmed or clinically suspected primary urological malignancy. Eligible designs were limited to prospective studies, including randomised controlled trials, prospective cohort studies, prospective diagnostic accuracy studies, and prospective registries with predefined outcome collection. All included studies had to involve intraoperative deployment of an AI-enabled or AI-adjacent system that directly informed surgical navigation, tissue assessment, or operative decision-making. Eligible technologies included real-time three-dimensional augmented or MR overlays, AI-assisted optical histology or pathology guidance, and intraoperative image registration systems providing real-time decision support.

Reports were required to describe at least one prespecified patient‑level clinical outcome (for example, perioperative complications, oncological margins, or functional recovery) or a diagnostic accuracy outcome benchmarked against a histopathological or clinical reference standard. Only full‑text English‑language articles published within the prespecified date range were included.

Retrospective studies of any design were excluded, as were animal, cadaveric, phantom, or simulation studies. Applications limited to preoperative planning, postoperative review, or education without intraoperative deployment were not eligible, nor were purely technical, algorithm‑development, or predictive‑modelling studies without intraoperative clinical use. Studies in non‑oncologic urological indications; conference abstracts, editorials, letters, reviews, and study protocols; and duplicate cohort reports (retaining only the most complete dataset) were excluded, as were publications outside the specified date window or not available as full‑text English articles.

One reviewer (GH) performed an intentionally over-inclusive title and abstract screening, which was independently double-checked by a second reviewer (BT) to confirm inter-rater reliability. All potentially eligible full-text articles were then assessed independently by both reviewers against the predefined criteria, with disagreements resolved by discussion and consensus. Data were extracted by one reviewer (GH) using a predefined extraction form and checked by a second reviewer (BT); discrepancies were resolved through discussion and consensus.

At screening, records suggesting possible intraoperative AI use or prospective design (including small feasibility studies, MR navigation, and preoperative AI segmentation with potential intraoperative application) were taken to full text to verify eligibility and characterise near-miss evidence. A small number of retrospective or technical studies were also reviewed in full to map the broader AI landscape and inform the discussion. Retrospective studies, conference abstracts, reviews, protocols, purely predictive or technical studies without intraoperative clinical use, and duplicates were excluded from the primary synthesis.

Data extraction

Data were extracted using a predefined framework capturing study design, setting, procedure, intervention, comparator, sample size, follow-up duration, and reported outcomes. Because the included literature comprised both therapeutic and diagnostic studies, extracted outcomes were grouped into oncological, functional, perioperative, technical, and diagnostic domains.

Risk-of-bias assessment

Risk of bias was assessed using design-specific tools appropriate to the included evidence base. The revised Cochrane Risk of Bias tool for randomised trials (RoB 2) was applied to randomised studies, ROBINS-I to non-randomised prospective interventional or cohort studies, and QUADAS-2 to prospective diagnostic accuracy studies [12-14]. This design-specific approach was chosen because the review included fundamentally different study types and a single generic checklist would have obscured rather than clarified methodological weaknesses.

Randomised studies were judged as having low risk of bias or some concerns only where key domains such as randomisation, deviations from intended intervention, missing data, measurement of outcome, and selective reporting appeared to be adequately handled in the published report [12]. Non-randomised studies were judged with particular attention to confounding, patient selection, intervention classification, deviations from intended intervention, missing data, outcome measurement, and selective reporting [13]. Diagnostic accuracy studies were assessed for patient selection, index test conduct and interpretation, reference standard quality, and flow and timing [14].

Data synthesis

Because of substantial heterogeneity in intervention type, mechanism of action, study design, comparator structure, and endpoint definition, no meta-analysis was performed. Findings were synthesised narratively and grouped by procedure type and technology class, with explicit separation between randomised comparative evidence, non-comparative interventional studies, and diagnostic validation studies.

Results

The search identified 131 records. All records underwent title and abstract screening, after which 93 were excluded. Thirty-eight record-level entries were carried forward for retrieval; after consolidation of duplicate database records referring to the same publications or underlying studies, these represented 22 unique reports. No report sought for retrieval was unavailable, and all 22 reports underwent full-text eligibility assessment. Sixteen reports were excluded at this stage for the reasons shown in Table 1. Six studies met the pre-specified eligibility criteria and were included in the narrative synthesis.

**Table 1:** Reports excluded at full-text eligibility assessment. Studies excluded after full-text review (*n* = 16). At screening, records suggesting possible intraoperative AI use or prospective design (including small feasibility studies, mixed-reality navigation, and preoperative AI segmentation with potential intraoperative application) were taken to full text to verify eligibility and characterise near-miss evidence. A small number of retrospective or technical studies were also reviewed in full to map the broader AI landscape and inform the discussion. Retrospective studies, conference abstracts, reviews, protocols, purely predictive or technical studies without intraoperative clinical use, and duplicates were excluded from the primary synthesis. 2D, two-dimensional; 3D, three-dimensional; 3D-AR, three-dimensional augmented reality; AI, artificial intelligence; BCR, biochemical recurrence; bpMRI, biparametric magnetic resonance imaging; C-index, centrality index; CSA, contact surface area; GTV, gross tumour volume; ICG, indocyanine green; ML, machine learning; MR, mixed reality; MRI, magnetic resonance imaging; NS-RARP, nerve-sparing robot-assisted radical prostatectomy; NVB, neurovascular bundle; PADUA, Preoperative Aspects and Dimensions Used for an Anatomical Classification; PCa, prostate cancer; PSM, propensity score matching; RAPN, robot-assisted partial nephrectomy; RARP, robot-assisted radical prostatectomy; RCC, renal cell carcinoma; USG, ultrasonography

Study	Study descriptor	Reason for full‑text inclusion	Reason for exclusion after full‑text review
Mei et al. [15]	AI segmentation of GTV and NVB on bpMRI to guide choice of intrafascial vs. extrafascial RARP; 40 patients prospectively included.	Prospective inclusion with AI‑assisted preoperative segmentation linked to surgical approach and functional outcomes; borderline intraoperative AI use.	AI used primarily for preoperative planning; intraoperative AI‑driven navigation/decision support not clearly demonstrated
Liu et al. [16]	Propensity score‑matched cohort of mixed‑reality navigation vs. standard RARP for high‑risk PCa.	Real‑time MR navigation reported intraoperatively with PSM and functional outcomes; design unclear from abstract.	Retrospective propensity‑matched cohort; does not meet prospective‑only criterion
Checcucci et al. [17]	3D‑AR‑guided selective NVB biopsy with real‑time VivaScope confocal pathology during RARP (*n* = 5; conference abstract).	Intervention closely matches intraoperative AI‑AR guidance with real‑time pathology; potential prospective feasibility study.	Conference abstract only; insufficient detail to confirm prospective design and predefined outcomes
Chen et al. [18]	Human‑in‑the‑loop validation of AI‑driven real‑time prostatic capsule recognition during NS‑RARP.	Real‑time AI anatomical guidance evaluated intraoperatively with surgeon ratings; potential clinical feasibility study.	Primary outcomes: technical (segmentation performance, acceptance ratings); no prespecified patient‑level clinical outcomes
Bang et al. [19]	Hybrid renorrhaphy with selective inner-layer clipping under AI‑assisted 3D navigation in RAPN (single case; conference abstract).	AI‑assisted 3D navigation used intraoperatively to guide vascular control and renorrhaphy; near‑miss technology.	Single‑case conference abstract; not a prospective clinical study
Angerer et al. [20]	Hyperaccuracy 3D model with intraoperative multimodal mixed‑reality visualisation for RAPN (single case; conference abstract).	Intraoperative mixed‑reality display of 3D model influencing clamping strategy; near‑miss technology.	Single‑case conference abstract; no prospective design or patient‑level outcomes
Passaro et al. [21]	Impact of 3D virtual modelling on perioperative outcomes in RAPN (3D‑assisted vs. standard; conference abstract).	Prospective data collection reported for 3D‑assisted RAPN with trifecta outcome; design unclear.	Conference abstract; quasi‑prospective/retrospective design; no explicit AI‑enabled intraoperative decision support
Boellaard et al. [22]	Comparison of AI‑assisted vs. manual prostate MRI segmentation towards 3D‑model‑guided prostatectomy.	AI segmentation proposed to enable 3D‑guided RARP; needed to confirm whether intraoperative use occurred.	Technical validation of segmentation only; no intraoperative AI deployment or clinical outcomes
Vermijs et al. [23]	Automated nephrometry scoring (RENAL, PADUA, C‑index, CSA) using 3D models in RCC patients undergoing RAPN.	AI‑enabled 3D models used in RAPN population; needed to confirm any intraoperative component.	Retrospective technical study; AI used only for preoperative nephrometry scoring
Grosso et al. [24]	3D virtual model‑assisted vs. 2D imaging for RAPN in totally endophytic tumours (propensity‑matched analysis).	3D guidance in complex RAPN with trifecta and margin outcomes; possible intraoperative navigation.	Retrospective analysis on a prospectively maintained dataset; no AI‑enabled intraoperative system
Margue et al. [25]	3D‑planned RAPN in horseshoe kidneys: European multicentre case series.	Complex RAPN cases using 3D reconstructions for vascular control; potential intraoperative guidance.	Retrospective case series; no AI‑enabled intraoperative decision support
Gupta et al. [26]	Combined use of ICG, intraoperative USG and 3D kidney model in complex RAPN.	Multimodal intraoperative guidance in RAPN; needed to confirm any AI component.	Retrospective series; 3D modelling used without AI‑enabled intraoperative decision support
Saikali et al. [27]	AI‑based ML models predicting continence and potency at 12 months after RARP (8,524 patients).	Large AI model in RARP population; needed to confirm any intraoperative element.	Retrospective predictive modelling; no intraoperative AI use
Rajih et al. [28]	ML‑based prediction of postoperative outcomes (BCR, PSM, functional) in 758 RARP cases.	ML models for key RARP outcomes; checked for any intraoperative application.	Retrospective predictive modelling; no intraoperative AI deployment
Fan et al. [29]	ML model predicting positive surgical margins after RARP (single‑centre, 301 patients).	PSM prediction in RARP cohort; assessed for intraoperative relevance.	Retrospective predictive model; no intraoperative AI use
Konnai et al. [30]	AI‑based surgical phase recognition in RARP with cross‑surgeon validation.	AI system recognising RARP phases; assessed for intraoperative decision support.	Retrospective video‑analytics; no intraoperative decision‑making or patient outcomes

The principal reasons for exclusion at full-text eligibility assessment were conference abstracts or non-peer-reviewed reports, retrospective or non-prospective designs, preoperative-only or non-intraoperative applications, studies without explicit AI-driven intraoperative deployment, case reports, technical or segmentation-only studies, and interventions unrelated to surgical navigation or pathology guidance (Table 1).

PRISMA flow diagram

The study flow is summarised in Figure 1.

**Figure 1:**
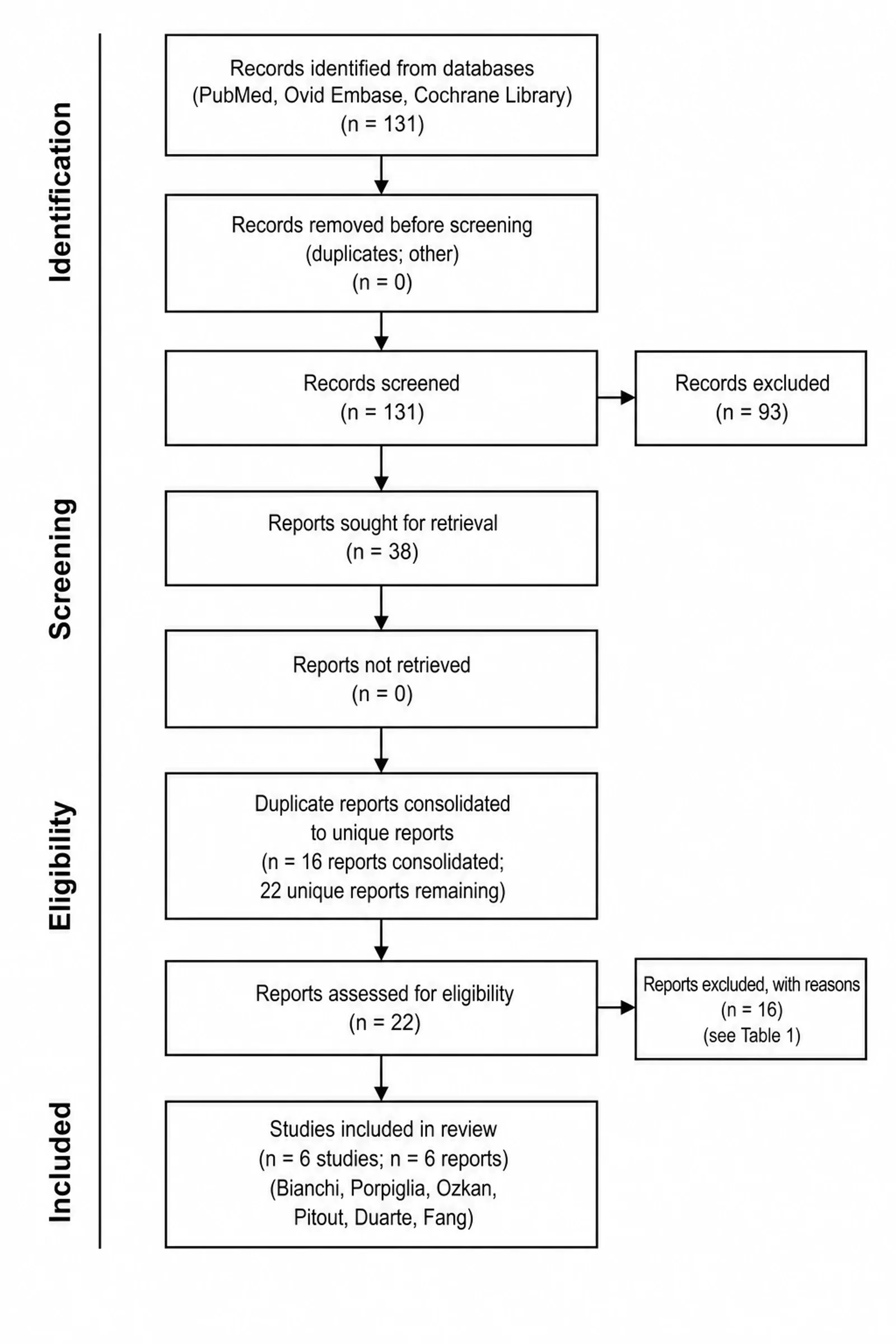
PRIMSA 2020 flow diagram of study identification and selection. The search yielded 131 records; 93 were excluded at title and abstract screening, 22 reports underwent full-text eligibility assessment after deduplication, 16 reports were excluded with reasons, and six studies, represented by six reports, were included in the review. PRISMA, Preferred Reporting Items for Systematic Reviews and Meta-Analyses

Study characteristics

Six prospective studies by Bianchi et al. [31], Porpiglia et al. [32], Özkan et al. [33], Pitout et al. [34], Duarte et al. [35], and Fang et al. [36] met the final inclusion criteria. Three studies involved robot-assisted radical prostatectomy [31-33], two involved robot-assisted partial nephrectomy [34,35], and one involved fluorescence confocal microscopy image interpretation during radical prostatectomy [36]. Two studies were randomised comparative trials [32,35], two were non-randomised prospective interventional or cohort studies [31,34], and two were prospective diagnostic accuracy studies (Table 2) [33,36].

**Table 2:** Characteristics of included studies. 3D, three-dimensional; AI, artificial intelligence; AR, augmented reality; AUC, area under the receiver operating characteristic curve; cryo-HE, cryosection haematoxylin and eosin; FCM, fluorescence confocal microscopy; MRI, magnetic resonance imaging; PLND, pelvic lymph node dissection; PSMA, prostate-specific membrane antigen; PSM, propensity score matching; RAPN, robot-assisted partial nephrectomy; RARP, robot-assisted radical prostatectomy; RP, radical prostatectomy; SRH, stimulated Raman histology

Study	Design	Procedure	Technology	Sample	Comparator	Main findings	Follow-up
Bianchi et al. [31]	Prospective single-institution study	RARP + PLND	AR-PSMA-3D	13	None	Sensitivity 69%, specificity 90%, AUC 0.79	Median 11 months
Porpiglia et al. [32]	Multicentre randomised trial	RARP	3D AI-AR	133	Cognitive MRI guidance	PSM 22% vs. 39%; postoperative radiotherapy 18% vs. 35%; total continence 91% vs. 71%	12-month functional follow-up
Özkan et al. [33]	Prospective pilot diagnostic study	RARP	AI-enhanced SRH	18	cryo-HE reference standard	Patient-based sensitivity 1.00, specificity 0.93	Intraoperative diagnostic study
Pitout et al. [34]	Prospective registry cohort	RAPN	3D image-guided surgery	568 patients/586 surgeries	None	Trifecta 55.2%; PSM 5.1%; renal preservation 59.9%	Mean 31.5 months
Duarte et al. [35]	Randomised trial	RAPN	Mixed reality	45	Standard RAPN	Warm ischaemia 14.6 vs. 18.4 min; selective clamping 25% vs. 0%	Short- and long-term renal follow-up reported
Fang et al. [36]	Prospective development and external validation study	RP specimen imaging	Deep learning FCM	24 patients in development cohort	Histopathology reference standard	Internal AUC 0.93; external AUC 0.83	Diagnostic validation

Risk of bias

Methodological quality varied across the included studies. The two randomised trials by Porpiglia et al. and Duarte et al. were judged to have some concerns overall on the RoB 2 assessment rather than low risk [32,35]. In both studies, randomisation was a major strength, but the available reports did not fully eliminate uncertainty regarding allocation concealment, practical blinding limitations, and completeness of outcome ascertainment for all clinically relevant endpoints. In Duarte et al., the small sample size further amplified imprecision and vulnerability to unstable effect estimates [35].

The non-randomised prospective studies by Bianchi et al. and Pitout et al. were judged to be at serious risk of bias using ROBINS-I [31,34]. The study by Bianchi et al. was a single-arm feasibility study in a highly selected population with no comparator and manual alignment of the 3D model, making confounding and selection bias likely [31]. Pitout et al. benefited from prospective registry capture and a large sample size, but the absence of a contemporaneous control group precluded robust causal inference regarding the benefit attributable to 3D image guidance itself [34].

The non-randomised prospective studies by Bianchi et al. and Pitout et al. were judged to be at serious risk of bias using ROBINS-I [31,34]. The study by Bianchi et al. was a single-arm feasibility study in a highly selected population with no comparator and manual alignment of the 3D model, making confounding and selection bias likely [31]. Pitout et al. benefited from prospective registry capture and a large sample size, but the absence of a contemporaneous control group precluded robust causal inference regarding the benefit attributable to 3D image guidance itself (Table 3) [34].

**Table 3:** Study-level risk-of-bias assessment. ROBINS-I, Risk Of Bias In Non-randomised Studies - of Interventions; RoB 2, Cochrane Risk of Bias 2; QUADAS-2, Quality Assessment of Diagnostic Accuracy Studies 2

Study	Tool	Overall judgement	Main domains of concern
Bianchi et al. [31]	ROBINS-I	Risk of bias	No comparator, selected cohort, confounding, operator-dependent registration
Porpiglia et al. [32]	RoB 2	Some concerns	Allocation/reporting details not fully resolved from publication; practical blinding limits
Özkan et al. [33]	QUADAS-2	High concern	Small pilot study, single centre, single-reader interpretation, limited applicability
Pitout et al. [34]	ROBINS-I	Risk of bias	No contemporaneous comparator, confounding by centre expertise and patient selection
Duarte et al. [35]	RoB 2	Some concerns	Small randomised study, practical blinding limits, imprecision
Fang et al. [36]	QUADAS-2	Moderate concern	Image-level rather than patient-level validation, limited external dataset applicability

Radical prostatectomy studies

Bianchi et al. prospectively enrolled 13 patients with high-risk prostate cancer and prostate-specific membrane antigen positron emission tomography (PSMA-PET)-detected nodal disease to undergo robot-assisted radical prostatectomy with AR prostate-specific membrane antigen three-dimensional (AR-PSMA-3D)-guided pelvic lymph node dissection. Four patients (30%) had suspicious nodal disease outside the standard pelvic lymph node dissection template, and nine patients (69%) had pathologically confirmed node-positive disease. On a per-region analysis of 117 dissected nodal regions, AR-PSMA-3D guidance yielded a sensitivity of 69%, specificity of 90%, positive predictive value of 52%, negative predictive value of 95%, and an area under the curve of 0.79. No intraoperative complications were reported [31].

Porpiglia et al. reported the RIDERS multicentre randomised controlled trial, which enrolled 133 patients with extracapsular extension or bulging on preoperative MRI who underwent nerve-sparing robot-assisted radical prostatectomy. The 3D AI-AR arm showed lower positive surgical margin rates than the cognitive MRI group, 22% versus 39%, and lower use of postoperative radiotherapy, 18% versus 35%. Twelve-month functional outcomes also favoured the 3D guidance group, with social continence rates of 94% versus 75% and total continence rates of 91% versus 71%, whereas potency and biochemical recurrence were reported as similar between groups. The positivity rate of neurovascular bundle excisional biopsies was higher in the 3D group, 52% versus 13%, consistent with a more selective real-time nerve-sparing strategy [32].

Özkan et al. reported preliminary findings from the prospective ROBOSPEC pilot study of AI-enhanced stimulated Raman histopathology for intraoperative margin assessment during robot-assisted radical prostatectomy. Eighteen patients were enrolled, 36 margin samples were collected, and 33 were evaluable after exclusion of three artefact-affected samples. NYU-AI identified positive surgical margins in four of 18 patients, corresponding to 22%, and patient-based analysis showed a sensitivity of 1.00, specificity of 0.93, positive predictive value of 0.75, and negative predictive value of 1.00. Sample-based analysis similarly showed a sensitivity of 1.00, specificity of 0.97, positive predictive value of 0.75, and negative predictive value of 1.00. The mean time for image generation was 8.2 minutes per sample [33].

Fang et al. developed and externally validated a deep-learning model for fluorescence confocal microscopy interpretation using material from the multicentre IP8-FLUORESCE study and an independent LaserSAFE dataset. The development cohort contained 275 images, including 37 tumour and 238 benign images from 24 patients, and internal testing was performed on 57 images. Internal performance included a sensitivity of 87.5%, specificity of 97.9%, positive predictive value of 87.5%, negative predictive value of 97.9%, and an AUC of 0.93. External validation on 46 independent images yielded a sensitivity of 91.3%, specificity of 73.9%, positive predictive value of 77.8%, negative predictive value of 89.5%, and an AUC of 0.83. The model generated predictions in under 2 seconds and was paired with a graphical user interface intended to support real-time intraoperative deployment [36].

Partial nephrectomy studies

Duarte et al. reported the REALITATEM randomised study comparing robot-assisted partial nephrectomy performed with and without MR guidance. In 45 patients, MR was associated with a shorter warm ischaemia time than standard surgery, 14.6 versus 18.4 minutes, and a higher rate of selective clamping, 25% versus 0%. No difference was observed in complications or in short- and long-term renal functional outcomes, indicating that the perioperative technical benefit did not clearly translate into postoperative functional superiority during the reported follow-up period [35].

Pitout et al. reported a large prospective cohort of 3D image-guided robot-assisted partial nephrectomy embedded in the UroCCR registry. The study included 568 patients undergoing 586 procedures at a tertiary referral centre, with prospectively collected clinical, biological, and radiological data. The primary endpoint, trifecta achievement, was defined as the absence of major complications, negative surgical margins in malignant tumours, and preservation of at least 90% of baseline renal function at 3 months. Trifecta was achieved in 55.2% of evaluable malignant cases, severe complications occurred in 5.6%, positive surgical margins in 5.1%, and renal function was preserved in 59.9% at 3 months. At a mean follow-up of 31.5 months, recurrence and metastasis rates were 7.4% and 8.6%, respectively, while cancer-specific survival and overall survival were 96.5% and 89%, respectively [34].

Overall synthesis

Across the six studies, comparative benefit was most convincingly demonstrated in the randomised trials [32,35]. In prostatectomy, the RIDERS trial provided the clearest signal that digital intraoperative guidance can influence both oncological and functional outcomes at the patient level [32]. In partial nephrectomy, MR improved selected technical endpoints, most notably warm ischaemia time and selective clamping, but without confirmed renal functional gain [35]. The remaining studies predominantly established feasibility, anatomical targeting, diagnostic performance, or medium-term descriptive outcomes rather than definitive effectiveness (Table 4) [31,33,34,36].

**Table 4:** Synthesis by technology class. AR, augmented reality; MR, mixed reality; AI, artificial intelligence

Technology class	Studies	Evidence signal	Key caveat
AR/MR navigation and visual guidance	[30,31,34]	Feasible and associated with selected oncologic, functional, or technical improvements	Registration fidelity, selected expert settings, limited standardisation
AI-assisted intraoperative pathology	[32,35]	Strong diagnostic promise for margin assessment	Diagnostic accuracy does not equal patient-level effectiveness
3D image-guided nephron-sparing surgery	[33]	Encouraging medium-term outcomes in a large cohort	No contemporaneous comparator

Discussion

This review identified a small but clinically meaningful body of prospective evidence on intraoperative digital guidance in robotic urological oncology. The most important finding is that the current literature should not be interpreted as evidence for a single intervention class. Instead, it comprises at least three distinct domains: intraoperative navigational guidance using AR or MR, AI-assisted optical pathology for margin assessment, and broader three-dimensional image-guided surgical planning with intraoperative application [8-10,31-36]. These categories differ in biological rationale, dependency on registration accuracy, operator interaction, and the nature of evidence required before clinical adoption.

The most persuasive patient-level evidence comes from the randomised prostatectomy study by Porpiglia et al. [32]. The trial suggests that real-time 3D AI-AR support may improve the balance between oncological safety and functional preservation during nerve-sparing surgery. This observation sits within a broader modern literature showing that intraoperative guidance strategies, when embedded in structured surgical workflows, can improve functional outcomes without necessarily worsening oncological control [3,4]. Broader prostatectomy literature also makes clear that improvements in early continence often do not automatically translate into durable long-term functional superiority, and that any continence-preserving strategy must still be judged against margin safety [2,4].

The partial nephrectomy evidence is more cautious. MR guidance appears capable of improving intraoperative decision-making around clamping strategy and reducing warm ischaemia time, which is clinically relevant because ischaemia duration remains a modifiable determinant of short-term postoperative renal function [6,7,35]. However, the absence of a clear postoperative renal functional advantage in Duarte et al. illustrates a broader problem in image-guided surgery research. Technical gains do not always translate into measurable patient-level benefit, particularly in small trials with limited follow-up [35]. The larger cohort reported by Pitout et al. provides useful reassurance regarding feasibility and medium-term outcomes in an experienced centre, but without a contemporaneous control group, it does not establish causal superiority of 3D image guidance over standard care [34].

The diagnostic studies deserve separate interpretation. Both Özkan et al. and Fang et al. demonstrated promising diagnostic performance for intraoperative margin assessment using optical histology platforms supported by AI [33,36]. The field is attractive because conventional frozen-section pathways are labour-intensive and not universally available, while recent randomised evidence in NeuroSAFE has shown that intraoperative pathology support can influence functional outcomes in prostatectomy when implemented within a formal surgical strategy [3]. Nonetheless, high sensitivity, specificity, or AUC should not be equated with proven clinical effectiveness. Diagnostic validation studies establish that a tool can classify tissue accurately under defined conditions; they do not prove that using the tool changes surgeon behaviour, reduces adjuvant treatment, improves continence or potency, or lowers recurrence risk in real-world practice [33,36].

A further contribution of this review is taxonomic rather than purely outcome-based. Several included studies were described by their authors as AI-enabled, yet the underlying clinical value often arose from augmented display, navigation, or workflow integration rather than from autonomous algorithmic recommendation [31,32,34,35]. This distinction is not semantic. It affects how clinicians interpret external validity, how reviewers assess the maturity of evidence, and how future trials should be designed. Studies of overlay navigation should report registration fidelity, intraoperative usability, and downstream clinical endpoints. AI pathology studies should report not only diagnostic performance but also reader interaction, failure modes, and any impact on surgical decision-making [14,33,36].

The present review and the underlying evidence base both have limitations. First, the review intentionally restricted inclusion to contemporary prospective studies published within a narrow date window, which improved topical relevance but inevitably omitted earlier developmental work. We wanted to review a modern snapshot of high-quality evidence to demonstrate how research is being undertaken in this space. Second, the evidence base was small and heterogeneous, preventing quantitative synthesis. Third, methodological quality varied considerably. The randomised studies were promising but still raised some concerns, whereas the non-randomised and diagnostic studies remained susceptible to confounding, selection bias, applicability limitations, and the common problem of extrapolating from feasibility to effectiveness [12-14]. Finally, most included studies were conducted in expert, technology-engaged centres, so generalisability to wider practice remains uncertain.

Future studies should proceed in a more structured way. Comparative trials should separate navigation systems from AI-assisted pathology rather than grouping them together under broad “AI surgery” terminology. Core outcomes should be standardised by procedure type. In radical prostatectomy, these should include positive surgical margins, adjuvant treatment, urinary continence, erectile function, and oncological follow-up. In partial nephrectomy, they should include warm ischaemia, selective clamping, renal function at prespecified time points, complications, and cancer control. Diagnostic studies should move beyond image-level accuracy and evaluate whether intraoperative deployment changes surgeon behaviour or patient outcomes [3,6,7,14].

## Conclusions

Intraoperative AI-related platforms have now entered prospective clinical evaluation in robot-assisted radical prostatectomy and partial nephrectomy. Across the small number of heterogeneous studies identified, these technologies appear technically feasible and safe, and they demonstrate promising signals in terms of surgical precision, functional preservation, and diagnostic performance. However, the current evidence base is limited by modest sample sizes, single-centre designs, and variability in both AI architectures and outcome definitions. At present, these systems should be regarded as emerging adjuncts rather than established standards of care in robotic urological oncology. Multicentre prospective trials with standardised reporting are needed to determine whether intraoperative AI-enabled guidance translates into durable oncological and functional benefit.
